# Supplementary material for: Quantifying local and global mass balance errors in physics-informed neural networks
Source: Sci Rep. 2024 Jul 5;14:15541. doi: 10.1038/s41598-024-65472-9 (PMC11229513; doi:10.1038/s41598-024-65472-9)
Supplement: Supplementary file 1 — Supplementary Information. [file 41598_2024_65472_MOESM1_ESM.pdf]

Supplementary material for 'Quantifying local and global mass balance errors in physics-informed neural networks'

M. L. Mamud[1†,*], M. K. Mudunuru[1], S. Karra[2], and B. Ahmmed[3]
[1]Subsurface Science Group, Pacific Northwest National Laboratory, Richland, WA 99352.
[*]Corresponding author: Md Lal Mamud, Email: lal.mamud@pnnl.gov
[†] Previously (graduate research assistant) at Earth and Environmental Sciences Division, Los Alamos National Laboratory, Los Alamos, NM 87545.
[†] Previously (graduate research assistant) at Geology and Geological Engineering, University of Mississippi, University, MS 38677.
[2]Environmental Molecular Sciences Laboratory, Pacific Northwest National Laboratory, Richland, WA 99352.
[3] Earth and Environmental Sciences Division, Los Alamos National Laboratory, Los Alamos, NM 87545.

1 Derivation of the numerical solutions

The one-dimensional steady-state balance of mass for groundwater flow in the absence of sources and sinks within the flow domain is:

$$\frac{\partial}{\partial x}\left[q(x)\right] = 0, \tag{s1}$$

where, $q(x)$, follows the Darcy's model given by :

$$q(x) = -K(x)\frac{\partial h}{\partial x}, \tag{s2}$$

where, $h[L]$ is the piezometric head, $x[L]$ is the coordinate, and $K[L/T]$ is the hydraulic conductivity.

Discretizing Eq. s1 gives us the mass balance equation across the faces of the nodes:

$$\frac{q_{i+\frac{1}{2}} - q_{i-\frac{1}{2}}}{dx} = 0$$
$$\text{or,} \quad q_{i+\frac{1}{2}} - q_{i-\frac{1}{2}} = 0$$

Using Eq. s2 in the above expression, we get:

$$\left[-K(x)\frac{d}{dx}h(x)\right]_{i+\frac{1}{2}} - \left[-K(x)\frac{d}{dx}h(x)\right]_{i-\frac{1}{2}} = 0$$
$$\text{or,} \quad -\left(\frac{K_{i+1}+K_i}{2}\right)\left(\frac{h_{i+1}-h_i}{\Delta x}\right) + \left(\frac{K_i+K_{i-1}}{2}\right)\left(\frac{h_i-h_{i-1}}{\Delta x}\right) = 0$$
$$\text{or,} \quad -\left(K_{i+1}+K_i\right)h_{i+1} + \left(K_{i+1}+K_i\right)h_i + \left(K_i+K_{i-1}\right)h_i - \left(K_i+K_{i-1}\right)h_{i-1} = 0$$
$$\text{or,} \quad -\left(K_{i+1}+K_i\right)h_{i+1} + \left(K_i+K_{i-1}+K_{i+1}+K_i\right)h_i - \left(K_i+K_{i-1}\right)h_{i-1} = 0$$

Therefore, the numerical solution for the heterogeneous medium for variable hydraulic conductivity, K can be written as follows:

$$(K_{i+1} + K_i)\, h_{i+1} - (K_{i+1} + 2K_i + K_{i-1})\, h_i + (K_i + K_{i-1})\, h_{i-1} = 0. \tag{s3}$$

The numerical solution in Eq. s3 can be modified for the homogeneous medium for constant hydraulic conductivity, K as follows:

$$(K + K)\, h_{i+1} - (K + 2K + K)\, h_i + (K + K)\, h_{i-1} = 0.$$
$$\text{or, } 2Kh_{i+1} - 4Kh_i + 2Kh_{i-1} = 0.$$

Therefore, the numerical solution for the homogeneous medium can be written as follows:

$$h_{i+1} - 2h_i + h_{i-1} = 0, \tag{s4}$$
